# Quercetin ameliorates ulcerative colitis by restoring the balance of M2/M1 and repairing the intestinal barrier via downregulating cGAS‒STING pathway

**DOI:** 10.3389/fphar.2024.1351538

**Published:** 2024-05-07

**Authors:** Fei Gao, Feng Zhu, Bo Shuai, Meng Wu, Chunzhu Wei, Yuyi Yuan, Yang Gui, Yushi Tian, Heng Fan, Hui Wu

**Affiliations:** ^1^ Department of Integrated Traditional Chinese and Western Medicine, Union Hospital, Tongji Medical College, Huazhong University of Science and Technology, Wuhan, China; ^2^ Department of Orthopedics, Union Hospital, Tongji Medical College, Huazhong University of Science and Technology, Wuhan, China

**Keywords:** ulcerative colitis, macrophage polarization, quercetin, intestinal barrier, immunity

## Abstract

Macrophage polarization is closely associated with the pathogenesis of ulcerative colitis (UC). Quercetin, a flavonoid, has shown promise as a treatment for inflammatory diseases, but its specific mechanism of action remains unclear. This study investigates whether quercetin can regulate intestinal macrophage polarization and promote intestinal tissue repair via the cGAS-STING pathway for the treatment of UC. *In vivo*, mice with 3% DSS-induced UC were intraperitoneally injected with quercetin and RU.521 for 7 days, following which their general conditions and corresponding therapeutic effects were assessed. The impact of interferon-stimulated DNA (ISD) and quercetin on macrophage polarization and the cGAS-STING pathway was investigated using RAW264.7 cells and bone marrow-derived macrophages (BMDMs) *in vitro*. The results demonstrated that ISD induced M1 macrophage polarization and activated the cGAS-STING pathway *in vitro*, while quercetin reversed ISD’s inflammatory effects. *In vivo*, quercetin suppressed the cGAS-STING pathway in the intestinal macrophages of DSS-induced UC mice, which reduced M1 macrophage polarization, increased M2 polarization, and facilitated intestinal barrier repair in UC. Taken together, these findings provide new insights into the mechanisms via which quercetin could be used to treat UC.

## 1 Introduction

Ulcerative colitis (UC), classified as an inflammatory bowel disease (IBD), presents with recurrent, prolonged and challenging-to-treat episodes. The intricate pathogenesis of UC increases the complexity of therapeutic approaches, which may lead to intestinal epithelial barrier disruption, immune response dysregulation, and disturbances in the gut microbiota (Costello et al., 2019; Li et al., 2022). Specifically, rectifying immune imbalances is a crucial aspect of UC treatment, with the restoration of the intestinal barrier representing a key therapeutic goal. Among the various immune cells within the intestinal environment, macrophages play a central role in innate immunity, engaging in functions such as cytokine secretion, pathogen elimination, modulation of inflammation, and tissue repair (Subramanian Vignesh et al., 2013; Park et al., 2017; Hoeffel et al., 2021; Virga et al., 2021). Therefore, comprehending the polarization of intestinal macrophages and the preservation of intestinal barrier integrity holds significant relevance for advancing the treatment of UC.

Macrophage polarization refers to the dynamic process by which macrophages transition into activated states under specific conditions (Murray, 2017). In normal physiological circumstances, intestinal macrophages phagocytose microorganisms and initiate a controlled immune response as antigen-presenting cells (Zhang et al., 2023). However, persistent and excessive inflammatory stimuli can lead to macrophage overactivation, which may damage the intestinal mucosa. During active phases of UC, macrophages tend to polarize towards an inflammatory phenotype known as M1, which may degrade tight junction (TJ) proteins, causing damage to the intestinal epithelial barrier and triggering excessive inflammation (Lissner et al., 2015). Comparatively, M2-type macrophages possess anti-inflammatory properties and contribute to tissue repair by reducing inflammation and promoting the healing of damaged tissues (Kim et al., 2021). Thus, the ratio of M2 to M1 macrophages can serve as an indicator of UC progression, while the integrity of the intestinal barrier reflects the capacity of macrophages to facilitate tissue repair following polarization.

Studies have reported abnormal increases in double-stranded DNA (dsDNA) expression in the serum and damaged intestinal tissues of patients with IBD and mice model. Cyclic guanosine monophosphate-adenosine monophosphate synthase (cGAS) plays a crucial role in recognizing cytoplasmic dsDNA, which can effectively activate cGAS (Dalekos et al., 1993; Hopfner and Hornung, 2020; Zhao et al., 2021). Specifically, cGAS detects the anomalous presence of dsDNA in the cytosol, leading to the synthesis of 2′3′-cyclic GMP-AMP (2′3′-cGAMP), a secondary messenger molecule, which subsequently binds to stimulator of interferon genes (STING) located in the endoplasmic reticulum, inducing a conformational change in STING. This altered conformation recruits TANK-binding kinase 1 (TBK1), initiating a cascade of phosphorylation events. Among the key substrates, interferon regulatory factor 3 (IRF3) undergoes phosphorylation and translocates to the nucleus, resulting in the induction of the expression of type I interferon (IFN), chemokines, and several other inflammatory mediators and pro-apoptotic genes (Decout et al., 2021; Zhang et al., 2022). When introduced as an exogenous dsDNA stimulus in macrophages, interferon-stimulated DNA (ISD) specifically activate the cGAS-STING pathway, leading to the polarization of macrophages towards the M1 phenotype. Notably, microglia display a shift towards the M2 phenotype upon cGAS knockout (Cao et al., 2018; Jiang et al., 2021). Consequently, it is of significant interest to investigate whether the modulation of the cGAS-STING pathway can mediate macrophage polarization in UC.

UC imposes a substantial economic burden on patients and national healthcare systems due to its prolonged disease course and the high cost of medications. Common therapeutic drugs, such as aminosalicylic acid, steroids, and immunosuppressants, can lead to adverse effects, including gastrointestinal discomfort, osteoporosis and bone marrow suppression. Quercetin (Que), a widely occurring natural flavonoid found in various plants, possesses important pharmacological properties, including antioxidant, antibacterial, anti-tumor and immune-regulatory activities. Importantly, Que exhibits low toxicity and minimal side effects (Zhou Y. et al., 2023). Numerous studies have identified Que as a potential therapeutic agent for UC due to its potent anti-inflammatory properties. However, the specific mechanisms underlying its efficacy remain poorly understood (Dicarlo et al., 2019; Gravina et al., 2023; Wang et al., 2024). In our study, we investigate whether Que can ameliorate UC by modulating macrophage polarization and promoting the restoration of the intestinal barrier through the cGAS-STING pathway.

## 2 Materials and methods

### 2.1 Drugs and reagents

Que (Cat#R006828) with a purity of HPLC ≥97% was purchased from Shanghai Rawhn Chemical Technology Co., Ltd. (Shanghai, China). RU.521 (Cat#M9447), a cGAS inhibitor, from AbMole (Houston, United States), DSS (Cat#160110) from MP Biomedicals (San Diego, CA, United States), FITC-Dextran (Cat#HY-128868D) from MedChemExpress (Monmouth Junction, NJ, United States), M-CSF (Cat#315-02) from PeproTech (Rocky Hill, NJ, United States). CCK-8 (Cat#BS350A) from Biosharp (Hefei, China), Lipofectamine™ 3000 (Lip3000, Cat#L3000001) and APC CD206 antibody (REF#17-2061-82) from Invitrogen (Carlsbad, CA, United States). iNOS antibody (Cat#18985-1-AP), Arg1 antibody (Cat#16001-1-AP), α-Tublin (Cat#66031-1-lg), STING (Cat#19851-1-AP), IRF3 (Cat#11312-1-AP), p-IRF3 (Cat#29528-1-AP), ZO1 (Cat#21773-1-AP) and Occludin (Cat#27260-1-AP) from Proteintech (Wuhan, China). Additionally, TBK1 (Cat#3504T) and p-TBK1 (Cat#5483S) were purchased from Cell Signaling Technology (Danvers, MA, United States), cGAS (Cat#ZRB1406) from Merck Millipore (Billerica, MA, United States), ELISA kits for IL10, CCL17, CXCL10, TNF-α and IFN-β from Adsbio (Yancheng, China), β-actin (Cat#GB12001), RIPA lysis buffer (Cat#G2002-100ML) and Bicinchoninic Acid (BCA) protein assay kit (Cat# G2026-200T) from Servicebio (Wuhan, China), and the following antibodies BV510 anti-FVD (Cat# 564406) and APC-Cy7 anti-CD45 (Cat# 557659), PE F4/80 (Cat#565410), PC-CY5.5 CD11b (Cat#101227) and BV421 CD11C (Cat#117329) from BD Biosciences (Franklin Lakes, NJ, United States). Furthermore, Trizol was bought from TaKaRa (Tokyo, Japan), HiScript^®^ III RT SuperMix for quantitative polymerase chain reaction (qPCR) (+gDNA wiper) and ChamQ SYBR qPCR Master Mix from Vazyme (Nanjing, China), blood urea nitrogen (BUN) assay kit (Cat#C013-2-1), creatinine (CRE) assay kit (Cat#C011-2-1), alanine aminotransferase (ALT) assay kit (Cat#C009-2-1) and aspartate aminotransferase (AST) assay kit (Cat# C010-2-1) from Jiancheng Bioengineering Institute (Nanjing, China).

ISD was generated by heating equimolar amounts of sense and antisense DNA oligonucleotides to 95°C for 5 min, followed by cooling to room temperature (Gui et al., 2019). Si-cGAS was synthesized by Paivi Biotechnology Co., Ltd. (Wuhan, China) and had the following sequences: si-cGAS (sense, 5′-CAG​CUG​AAC​ACU​GGC​AGC​UAC​UAU​G-3′, antisense, 5′-CAU​AGU​AGC​UGC​CAG​UGU​UCA​GCU​G-3′). All oligonucleotides for ISD and qPCR primers used in this study were provided by Beijing Tsingke Biotech Co., Ltd.

### 2.2 Mice

Male C57BL/6 J mice (weighing 20–22 g and aged 6–8 weeks) were purchased from SPF Biotechnology Co., LTD. (Beijing) (Quality certification: SCXK (jing) 2019-0010). All animal-related experiments were conducted in strict adherence to the guidelines of the Animal Research Institute Committee of HUST and were approved by the HUST Institutional Animal Care and Use Committee (IACUC).

The mice were provided unrestricted access to food and water and housed under specific pathogen-free (SPF) conditions, which included a 12-h light/dark cycle, a temperature maintained at 22 ± 1°C, and humidity levels ranging from 45% to 55%. They were allowed to acclimate for 5 days, after which they were divided into four groups (*n* = 8): the normal control group (Normal), the model group (DSS), the RU.521 group (RU.521, administered at a dosage of 10 mg/kg, serving as the positive control) (Ma et al., 2020), and the Que group (QUE, administered at a dosage of 30 mg/kg) (Yang et al., 2014). Except for the control group, acute colitis was induced by the administration of 3% DSS for 7 days. RU.521 and Que were administered via intraperitoneal injections over the same seven-day period, starting at the onset of colitis induction. Meanwhile, the model group received an equivalent volume of drinking water. On the eighth day, the mice were euthanized, and the tissues were collected for analysis.

### 2.3 Cells

RAW264.7 cells (Cat#SNL-112) were purchased from SUNNCELL (Wuhan, China) and cultured in Dulbecco’s Modified Eagle’s Medium (DMEM) with high glucose containing 5% fetal bovine serum (FBS). The culture was maintained in a humidified incubator with 5% CO2 and 95% air at 37°C. Bone marrow mesenchymal cells were isolated from the tibiofibula of mice and were stimulated with M-CSF at a concentration of 30 ng/mL in 5% FBS-1640 RPMI medium. The medium was replaced with fresh medium containing 20 ng/mL M-CSF on the third and fifth days. On the seventh day, the cell morphology was observed and confirmed through flow cytometry.

To determine the appropriate concentration of Que for intervention, the viability of both cell types was assessed after exposure to different concentrations of Que for 24 h, following the instructions provided with the CCK8 kit.

The cell experiment involved four distinct groups: the control group, the ISD (2 μg/mL) group, the si-cGAS + ISD group, and the Que (50 μmol/L) + ISD group. RAW264.7 cells and BMDMs were seeded in 9.6 cm^2^ culture plates at a density of 5 × 10^5^ cells per well and allowed to grow for 12 h in a humidified incubator. Subsequently, cells in all groups, except the control group, were transfected with ISD for 6 h. Following this transfection, the culture medium for all groups was replaced with 2 mL of fresh medium. Que was administered for an additional 24 h, and si-cGAS was transfected during the final 8 h of this period. To confirm the efficiency of si-cGAS transfection, quantitative real-time polymerase chain reaction analysis (qRT-PCR) and western blotting (WB) were performed. The transfection of ISD and si-cGAS was performed following the instructions provided for Lipofectamine™ 3000.

### 2.4 Evaluation of intestinal inflammation in mice

Throughout the experimental period, the weight of each mouse was consistently monitored. On the eighth day following drug intervention, the entire colon tissue was excised, photographed to record its length, and collected after mice were humanely euthanized via cervical dislocation under anesthesia induced by 1% pentobarbital sodium (50 mg/kg body weight). The intestines, specifically a segment approximately 0.5 cm proximal to the anus, were rinsed, circumferentially cut, and then preserved in 1 mL of 4% paraformaldehyde for the following experiments. To assess disease activity and calculate the disease activity index (DAI), weight loss, stool consistency and fecal occult blood were evaluated daily (Sann et al., 2013). Tissue sections were subjected to hematoxylin and eosin (H&E) staining for histological examination, while Alcian Blue/Phosphoric Acid Schiff (AB/PAS) staining was employed to visualize goblet cells, as previously described by Ma et al. (2020). Additionally, H&E staining was performed on the mice heart, liver, spleen, lung, and kidney tissues. The levels of blood urea nitrogen (BUN), creatinine (CRE), alanine aminotransferase (ALT) and aspartate aminotransferase (AST) in mouse serum were measured using assay kits to assess the safety of Que intervention in this study.

### 2.5 Preparation for single-cell suspension of mouse spleen, mesenteric lymph node

The procedure for obtaining a single-cell suspension from the mouse spleen and mesenteric lymph nodes was performed as previously described (Gao et al., 2022). Upon collection of the spleen and mesenteric lymph nodes, they were promptly immersed in pre-chilled 3% BSA in PBS. The tissues were finely chopped and softly homogenized. Following filtration through a 70 μm sieve, the cellular suspension was harvested. For spleen samples, erythrocytes were lysed prior to centrifugation and subsequent resuspension.

### 2.6 Flow cytometry

Flow cytometry was utilized to identify BMDMs and assess the M2/M1 cell ratio in the spleen, and MLNs post-intervention for macrophage polarization analysis. On the seventh day of M-CSF stimulation, bone marrow mesenchymal cells were collected and labeled with antibodies, including anti-FVD, APC-Cy7-conjugated anti-CD45, PE-conjugated F4/80, and PC-CY5.5-conjugated CD11b. For macrophage identification, cultured BMDMs received BV421-conjugated CD11C and APC-conjugated CD206 antibodies. Macrophages were characterized as F4/80^+^CD11b^+^ cells, with M1 and M2 subsets defined as F4/80^+^CD11b^+^CD11C^+^CD206^-^ and F4/80^+^CD11b^+^CD11C^−^CD206^+^, respectively. The same flow cytometry protocol was applied to assess macrophage polarization in tissues (Ying et al., 2013; Ding et al., 2022). Cells and other tissues were analyzed using a BD LSR flow cytometer (BD Biosciences, San Jose, CA, United States), and the data were analyzed using the CytExpert software.

### 2.7 qRT-PCR

Total RNA was extracted from both clones and cells using Trizol. Subsequently, reverse transcription was carried out using HiScript^®^ III RT SuperMix for qPCR (+gDNA wiper). Following reverse transcription, qPCR was conducted using ChamQ SYBR qPCR Master Mix. The procedures were conducted following the instructions of the respective reagents. The relative mRNA expression levels were calculated using the 2^−ΔΔCT^ method, with β-actin serving as the endogenous control. The primer sequences are provided in Table 1.

**TABLE 1:** Primers sequences.

Gene	Forward primer (5′–3′)	Reverse primer (5′–3′)
ISD	TAC​AGA​TCT​ACT​AGT​GAT​CTA​TG	ACT​GAT​CTG​TAC​ATG​ATC​TAC​A
cGAS	TTC​CAC​GAG​GAA​ATC​CGC​TGA​G	CAG​CAG​GGC​TTC​CTG​GTT​TTT​C
STING	GGT​GGC​CAG​CCT​GAT​GAT​CC	AGC​CTT​CCA​GTA​GCT​GCC​CT
iNOS	GAA​GAA​AAC​CCC​TTG​TGC​TG	TCC​AGG​GAT​TCT​GGA​ACA​TT
Arg1	CTT​GGC​TTG​CTT​CGG​AAC​TC	GGA​GAA​GGC​GTT​TGC​TTA​GTT​C
IFN-β	CTA​ACT​GCA​ACC​TTT​CGA​AGC	CTA​GTG​TCC​TTT​CAT​ATG​CAG
CCL17	CGA​GAG​TGC​TGC​CTG​GAT​TAC​T	GGT​CTG​CAC​AGA​TGA​GCT​TGC​C
IL10	GCT​CTT​ACT​GAC​TGG​CAT​GAG	CGC​AGC​TCT​AGG​AGC​ATG​TG
TNF-α	TAC​TGA​ACT​TCG​GGG​TGA​TCG	TCC​TCC​ACT​TGG​TGG​TTT​GC
CXCL10	CCA​AGT​GCT​GCC​GTC​ATT​TTC	TCC​CTA​TGG​CCC​TCA​TTC​TCA
ZO1	CCA​GCA​ACT​TTC​AGA​CCA​CC	TTG​TGT​ACG​GCT​TTG​GTG​TG
Occludin	TAA​GAG​CTT​ACA​GGC​AGA​ACT​AG	CTG​TCA​TAA​TCT​CCC​ACC​ATC

### 2.8 Western blotting

The cells or intestinal tissues were homogenized in RIPA lysis buffer using a grinder (50Hz, 120s, LUKA, Guangzhou). After centrifugation, the supernatant was collected, and protein concentrations were determined using a BCA protein assay kit. Subsequently, the samples were loaded onto either 8% or 10% sodium dodecyl sulfate–polyacrylamide gel electrophoresis (SDS-PAGE) gels with 15 wells and a thickness of 1.0 mm for rapid electrophoresis (200V, 30min). The target proteins were then transferred to a polyvinylidene (PVDF) membrane (Millipore, Billerica, MA, United States) (400mA, 30 min). Following a 20-min blocking step with a fast-blocking solution at room temperature, the protein bands were washed with 1 × TBST for 10 min and subsequently incubated with primary antibodies including cGAS, STING, TBK1, p-TBK1, iNOS, p-IRF3, β-actin, α-Tubulin (at a dilution of 1:1000), IRF3, and Arg1 (at a dilution of 1:5000) overnight at 4°C. The next day, the protein bands were washed three times with 1×TBST for 10 min each time and incubated with a secondary antibody (dilution, 1:5000) at room temperature for 1 h, followed by additional washing. Lastly, the bands were briefly immersed in an ECL exposure solution and placed in an exposure apparatus for visualization. Image analysis was performed using ImageJ software.

### 2.9 Enzyme-linked immunosorbent assay (ELISA)

The expression levels of IL10, CCL17, CXCL10, TNF-α and IFN-β in the colon homogenate supernatant were assessed using ELISA kits. Following the manufacturer’s instructions, the standard product was initially diluted in a gradient, then both the samples and biotin-labeled antibodies were introduced to the enzyme labeling plate. Absorbance was measured at 450 nm using a microplate reader.

### 2.10 Immunofluorescent (IF)

Intestinal sections stained with H&E were deparaffinized, while RAW264.7 cells were fixed using 4% paraformaldehyde. After rehydration, both were washed with 1% TWEEN-PBS and blocked with 3% goat serum. For IF experiments, specific primary antibodies (anti-iNOS, anti-ZO1, anti-Occludin at 1:200 dilution, and anti-Arg1 at 1:500 dilution) were incubated overnight at 4°C after blocking. On the following day, the sections and cells were washed and subsequently incubated with secondary antibodies labeled with fluorescein isothiocyanate (FITC) or Cy3. Finally, they were counter-stained with 4′,6-diamidino-2-phenylindole (DAPI) for 5 min, and IF images were captured using a confocal laser scanning microscope (Olympus-FV1000).

### 2.11 FITC-dex assay

The abdominal hairs of the mice were removed, and they underwent a 24-h fasting. Next, they were orally administered with FITC-Dex (average molecular weight: 4000, 0.6 mg/g) while being kept away from light for 4 h. Prior to the assay, the mice were anesthetized using 1% pentobarbital. To measure fluorescence intensity (excitation 490 nm/emission 535 nm), serum samples were diluted 1:1 with a PBS solution. The quantification of Fluorescein isothiocyanate-dextran (FITC-Dex) in the serum was performed using a fluorescent enzyme label kit, following the manufacturer’s instructions.

### 2.12 Statistical analysis

Statistical analysis was conducted using GraphPad Prism 8.0 (Graph Pad Software Inc., San Diego, CA, United States). Data are presented as mean ± standard deviation (SD). Two-tailed unpaired t-tests were employed to compare two groups, while a two-way analysis of variance (ANOVA) was utilized to analyze multiple groups. The significance levels were denoted as *p < 0.05, **p < 0.01, and ***p < 0.001.

## 3 Results

### 3.1 Quercetin ameliorates colitis symptoms and damage in colon tissues

**FIGURE 1:**
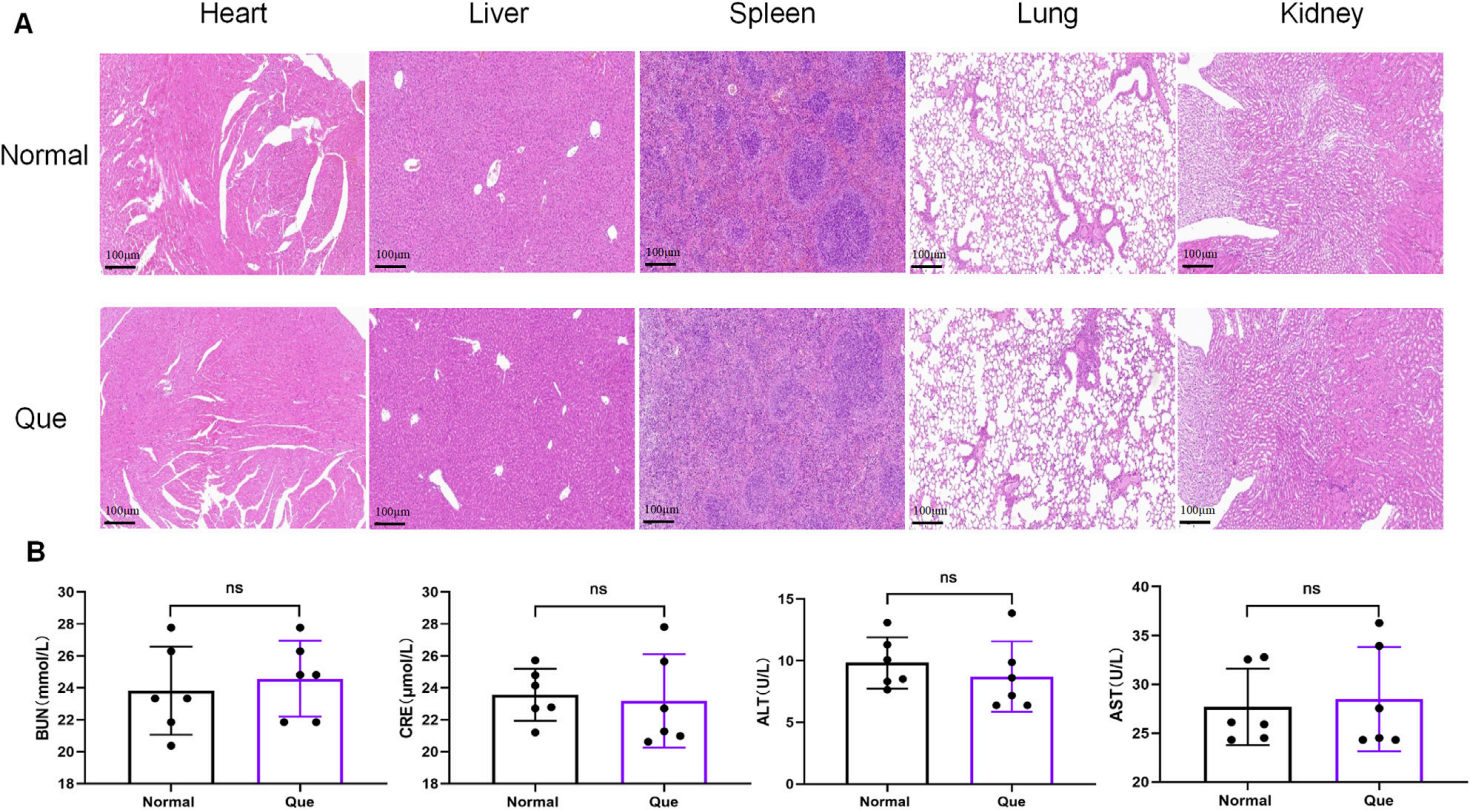
Quercetin is safe for mice. **(A)**. HE staining of heart, liver, spleen, lung, kidney tissues (Bar = 100 μm) (*n* = 6) **(B)** Statistical map of the levels of BUN, CRE, ALT and AST in mouse serum (*n* = 6). Data were shown as mean ± SD. ns (no significance) vs. normal group.

**FIGURE 2:**
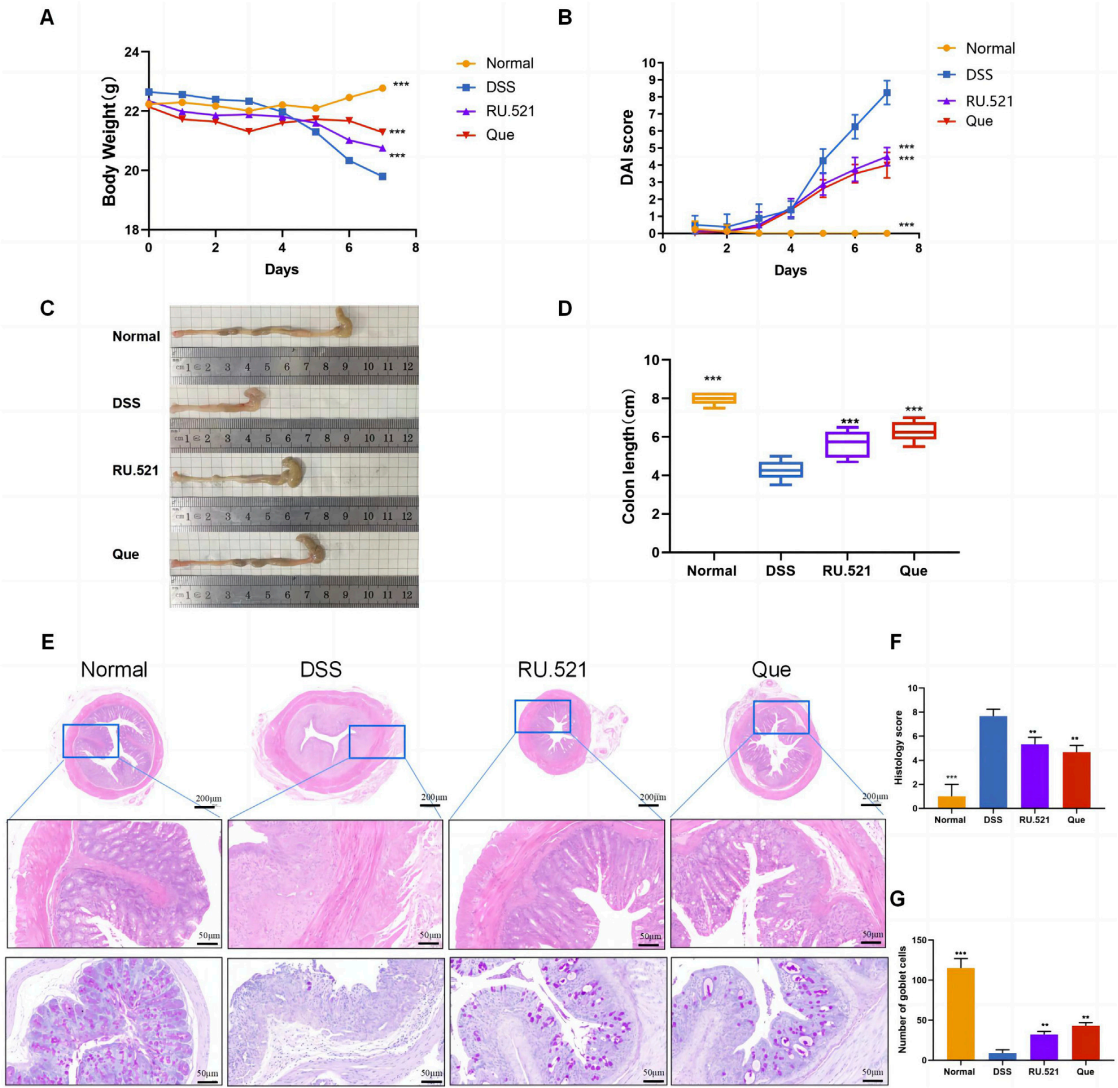
Quercetin alleviates DSS-induced UC. **(A)** Changes in body weight (*n* = 8). **(B)** DAI scores (*n* = 8). **(C)** Representative images of colon specimens from each group (*n* = 6). **(D)** Statistical analysis of colon length (*n* = 6). **(E)** Histological examination of colon tissues by HE and PAS staining (Bar = 200 μm, 50 μm) (*n* = 3). **(F)** Colonic histopathological scoring. **(G)** Number of goblet cells. Data are presented as mean ± SD. **p < 0.01,***p < 0.001.

The DSS-induced UC method in mice is well-established. First, we verified that Que at a concentration of 30 mg/kg was safe for mice after 7 days of intervention, The results of H&E staining showed that no obvious changes in the heart, liver, spleen, lung, or kidney tissues between the normal and Que-treated groups of mice. In addition, there were no significant deviations in serum levels of BUN, CRE, ALT and AST between groups (Figure 1). As shown in Figures 2A, B, mice treated with the cGAS inhibitor (RU.521) and Que exhibited significant improvements in weight loss, fecal characteristics and overall wellbeing compared to those receiving only DSS. Moreover, the interventions led to significant improvements in intestinal morphology and pathology, including reduced intestinal shortening, decreased infiltration of inflammatory cells, preserved crypts, and increased goblet cell counts in DSS-treated mice (Figures 2C–G). These results highlight the effectiveness of cGAS inhibition in reducing intestinal damage and improving UC and support the therapeutic potential of Que.

### 3.2 Quercetin restores M2/M1 balance in mice

**FIGURE 3:**
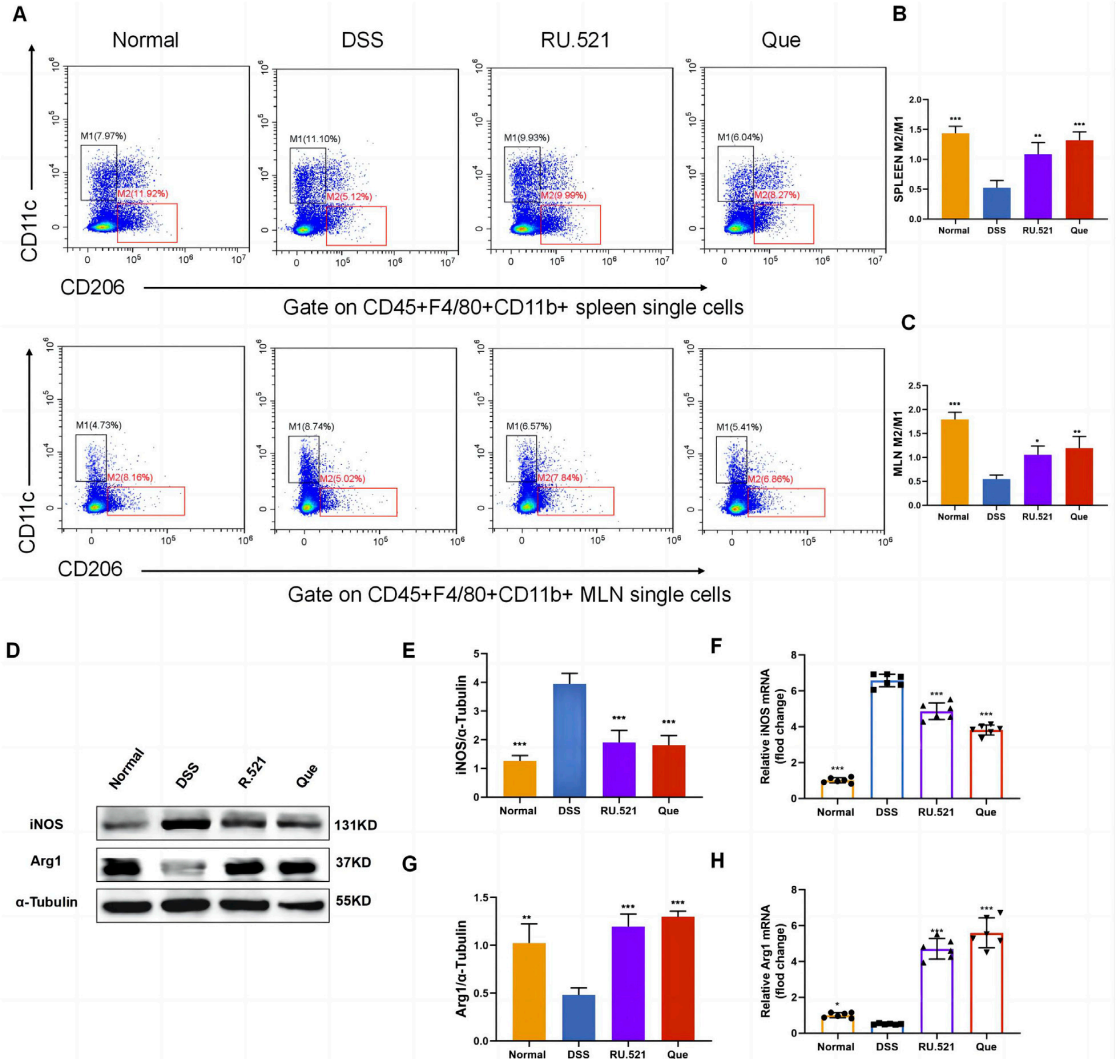
Quercetin restores M2/M1 ratio in mice. **(A)** Representative flow cytometry results and summarized percentages of CD11c^+^CD206^-^ cells (M1) and CD11c^−^CD206^+^ cells (M2) in the CD45^+^F4/80^+^CD11b^+^ macrophages of spleen and mesenteric lymph nodes. **(B)** Statistical analysis of M2/M1 ratio in mice spleen (*n* = 3). **(C)** Statistical analysis of M2/M1 ratio in mice mesenteric lymph nodes (*n* = 3). **(D)** Representative western blot images of iNOS and Arg1 in mice colon tissues (*n* = 3). **(E,G)** Statistical analysis of iNOS and Arg1 protein abundance in mice colon (*n* = 3). **(F,H)** Statistical analysis of iNOS and Arg1 mRNA abundance in analysis colon (*n* = 6). Data are shown as mean ± SD. *p < 0.05, **p < 0.01, ***p < 0.001.

To investigate the changes in M2 and M1 subpopulations in mice following Que intervention and cGAS inhibition, we isolated the spleen and mesenteric lymph nodes for flow analysis. Compared to the model group, the intervention groups exhibited significant suppression of the M1 subpopulation and an increase in the M2 subpopulation (Figures 3A–C). Furthermore, the levels of Arg1 and iNOS in intestinal tissues were assessed using WB and qPCR. It was observed that iNOS levels increased while Arg1 levels decreased during colitis. However, these changes were reversed following intervention with RU.521 and Que (Figures 3D–H). Thus, these findings indicate that Que and cGAS inhibition contribute to the restoration of the M2/M1 ratio in mice.

### 3.3 Intervention concentration of quercetin for successfully inducing BMDMs

**FIGURE 4:**
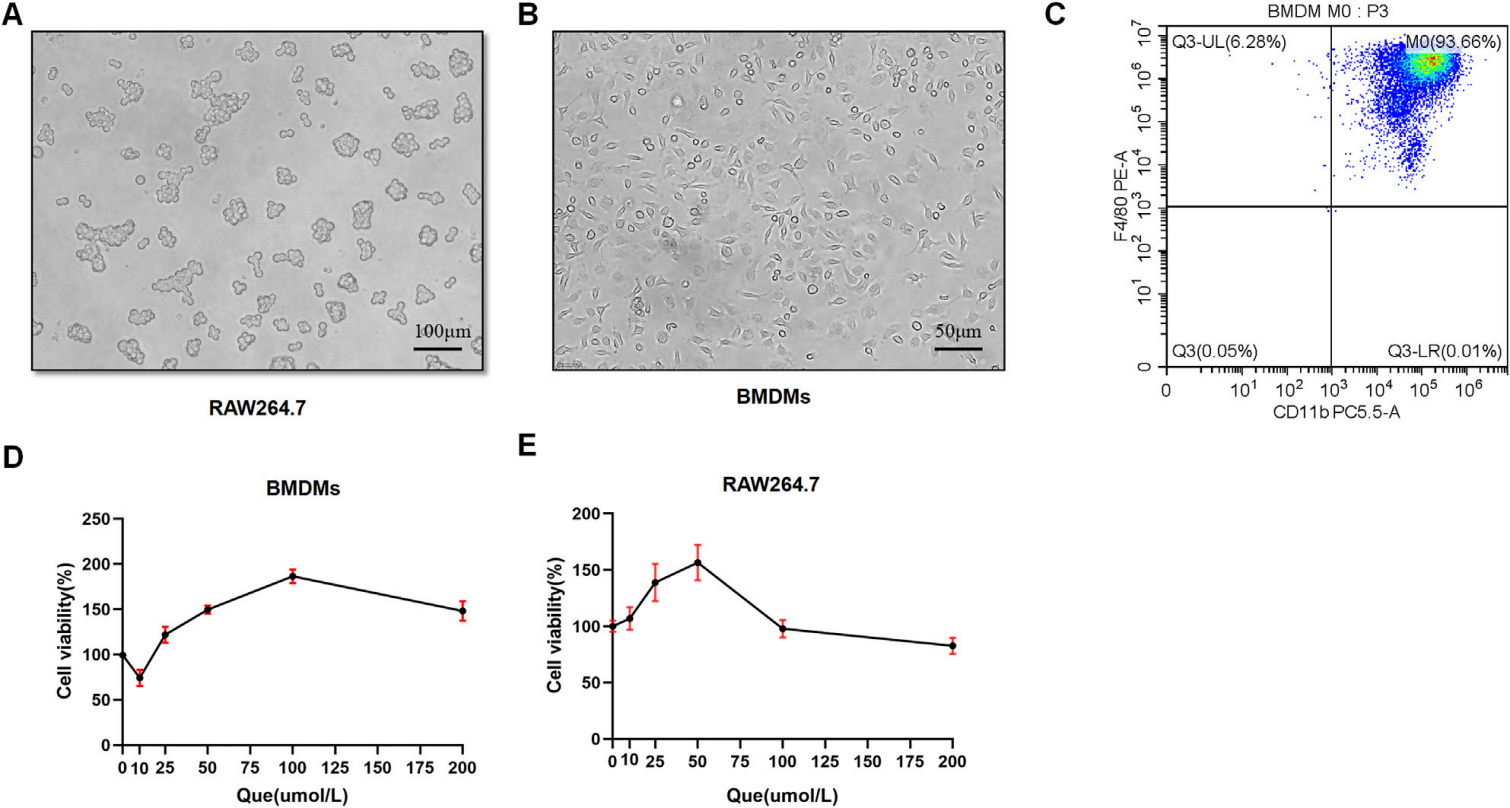
Successful induction of BMDMs and macrophage viability after 24-h quercetin (Que) intervention. **(A)** Morphology of RAW264.7 observed under a light microscope (Bar = 100 μm). **(B)** Morphology of BMDMs observed under a light microscope (Bar = 50 μm). **(C)** Flow cytometry results confirm the identification of BMDMs. **(D)** Viability of BMDMs. **(E)** Viability of RAW264.7 cells. Data are presented as mean ± SD.

BMDMs and RAW264.7 cells were used to assess macrophage polarization *in vitro*. Initially, we observed the morphology of RAW264.7 cells with round shape, and the M-CSF-induced cells which displayed a flattened appearance with numerous protrusions (Figures 4A, B). Subsequently, flow cytometry analysis revealed that F4/80^+^CD11b^+^ cells constituted 93.66% of the total cells, confirming successful macrophage induction (Figure 4C). BMDMs and RAW264.7 cells were subjected to different concentrations of Que for 24 h, and 50 μmol/L was determined as the optimal intervention concentration for Que, as it did not affect the viability of both cell types (Figures 4D, E).

### 3.4 Quercetin regulates macrophage polarization *in vitro*

**FIGURE 5:**
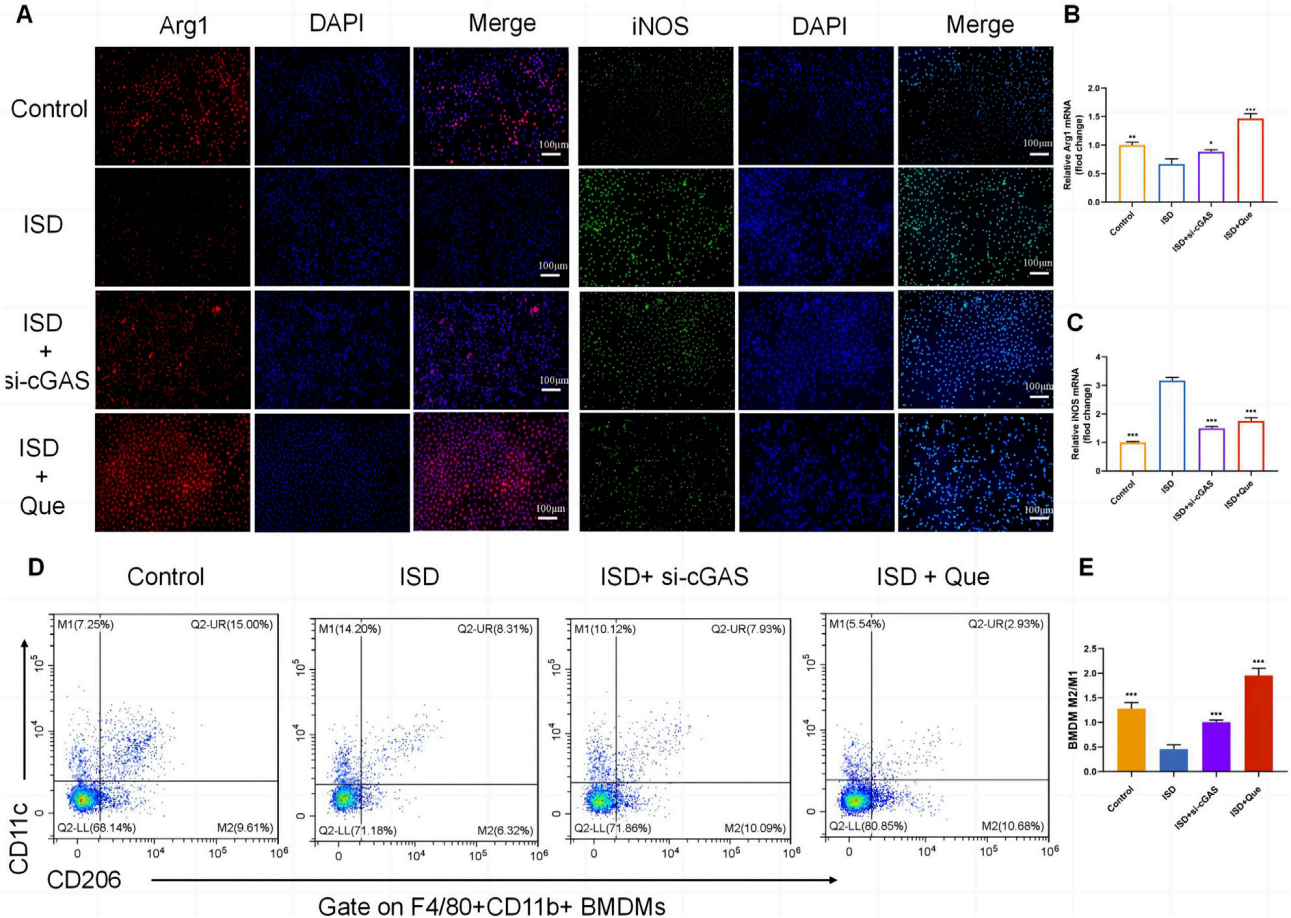
Quercetin restores the ratio of M2/M1 *in vitro*. **(A)** Immunofluorescence staining of Arg1 (red) and iNOS (green) expression in BMDMs (Bar = 100 μm); Nuclei were stained with DAPI (blue). **(B,C)** Quantification of iNOS and Arg1 mRNA levels in RAW264.7 cells (*n* = 3). **(D)** Flow cytometry results showing the percentages of M1 and M2 macrophages in BMDMs. **(E)** Statistical analysis of the M2/M1 ratio in BMDMs (*n* = 3). Data are presented as mean ± SD. *p < 0.05, **p < 0.01, ***p < 0.001 vs. ISD group.

To investigate the impact of Que and cGAS inhibition on macrophage polarization *in vitro*, we used ISD, an exogenous stimulator known to activate cGAS expression, to intervene with macrophages. We assessed the state of macrophage polarization after inhibiting cGAS expression and administering Que. Following ISD intervention, there was a significant decrease in the fluorescence expression of Arg1 and an increase in iNOS in RAW264.7 cells. However, when compared to the ISD group, the utilization of si-cGAS and Que led to a significant enhancement in Arg1 fluorescence expression, accompanied by a reduction in iNOS levels (Figure 5A). Similar changes were observed in the mRNA levels of Arg1 and iNOS in RAW264.7 cells (Figures 5B, C). Flow cytometry analysis provided further insights, demonstrating that ISD intervention resulted in an increased proportion of M1 cells and a reduced population of M2 cells. However, si-cGAS and Que effectively reversed this trend, redirecting macrophages away from the inflammatory phenotype (M1) towards M2 cells with anti-inflammatory properties (Figures 5D, E).

### 3.5 Quercetin inhibits the cGAS-STING pathway during macrophage polarization

**FIGURE 6:**
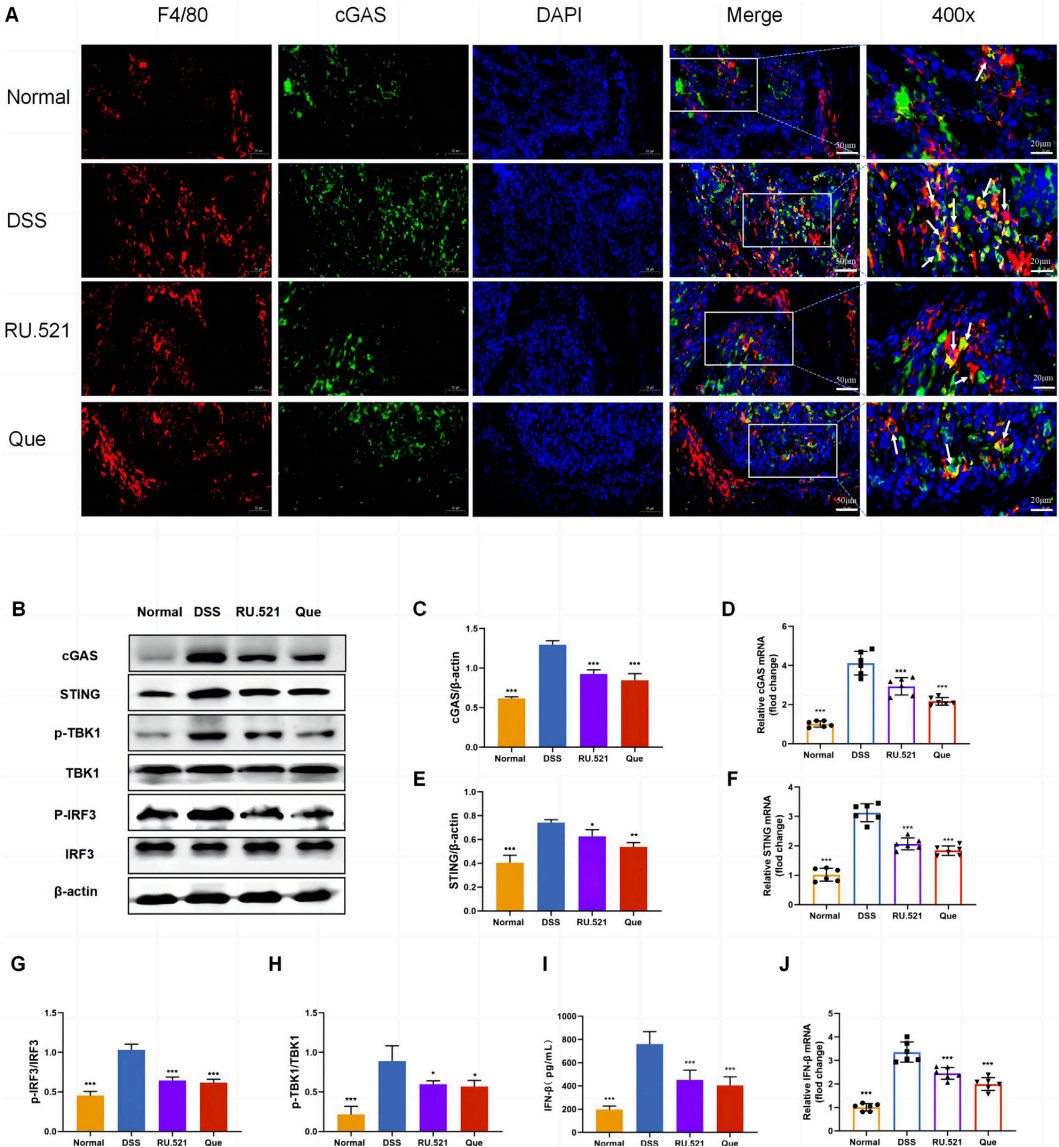
Quercetin inhibits the cGAS-STING pathway in intestinal macrophages. **(A)** Immunofluorescence staining of F4/80 (red) and cGAS (green) expression in colon tissues (Bar = 50 μm), with an enlarged view at 400× (Bar = 20 μm). Nuclei were stained with DAPI (blue). **(B)** Western blot analysis of the cGAS-STING pathway in colon tissues (*n* = 3). **(C,E,G,H,I)** Quantification of cGAS, STING, p-TBK1, p-IRF3 and IFN-β protein levels in colon tissues (*n* = 6). **(D,F,J)** Quantification of cGAS, STING, and IFN-β mRNA levels in colon (*n* = 6). Data are presented as mean ± SD. *p < 0.05, **p < 0.01, ***p < 0.001.

To investigate the role of the cGAS-STING pathway in the polarization of intestinal macrophages, we conducted double fluorescent labeling of intestinal F4/80 and cGAS proteins, which helped identify macrophages in the intestine. We observed that both Que and RU.521 reduced the fluorescence expression of F4/80 compared to the model group, indicating a decrease in intestinal macrophage infiltration. Under a 400-fold fluorescence microscope, we observed that the fluorescence expression of cGAS in intestinal macrophages was elevated in the intestinal tissues of mice in the model group. However, this expression was significantly reduced after the intervention of Que and RU.521, suggesting that both Que and RU.521 inhibited cGAS in intestinal macrophages (Figure 6A). Furthermore, we examined the changes in the cGAS-STING pathway in the colon and found that the expression of cGAS-STING pathway-related proteins (cGAS, STING, p-TBK, p-IRF3 and IFN-β) in mice significantly increased after modeling. Both Que and RU.521 reduced the levels of these proteins (Figures 6B, C, E, G–I). Additionally, mRNA levels of cGAS, STING, and IFN-β were detected by qPCR in both *in vitro* and *in vivo* experiments (Figures 6D, F, J). Based on findings regarding the regulation of macrophage polarization by Que and cGAS inhibition, it is evident that Que can inhibit the cGAS-STING pathway in gut macrophages, thereby influencing macrophage polarization.

**FIGURE 7:**
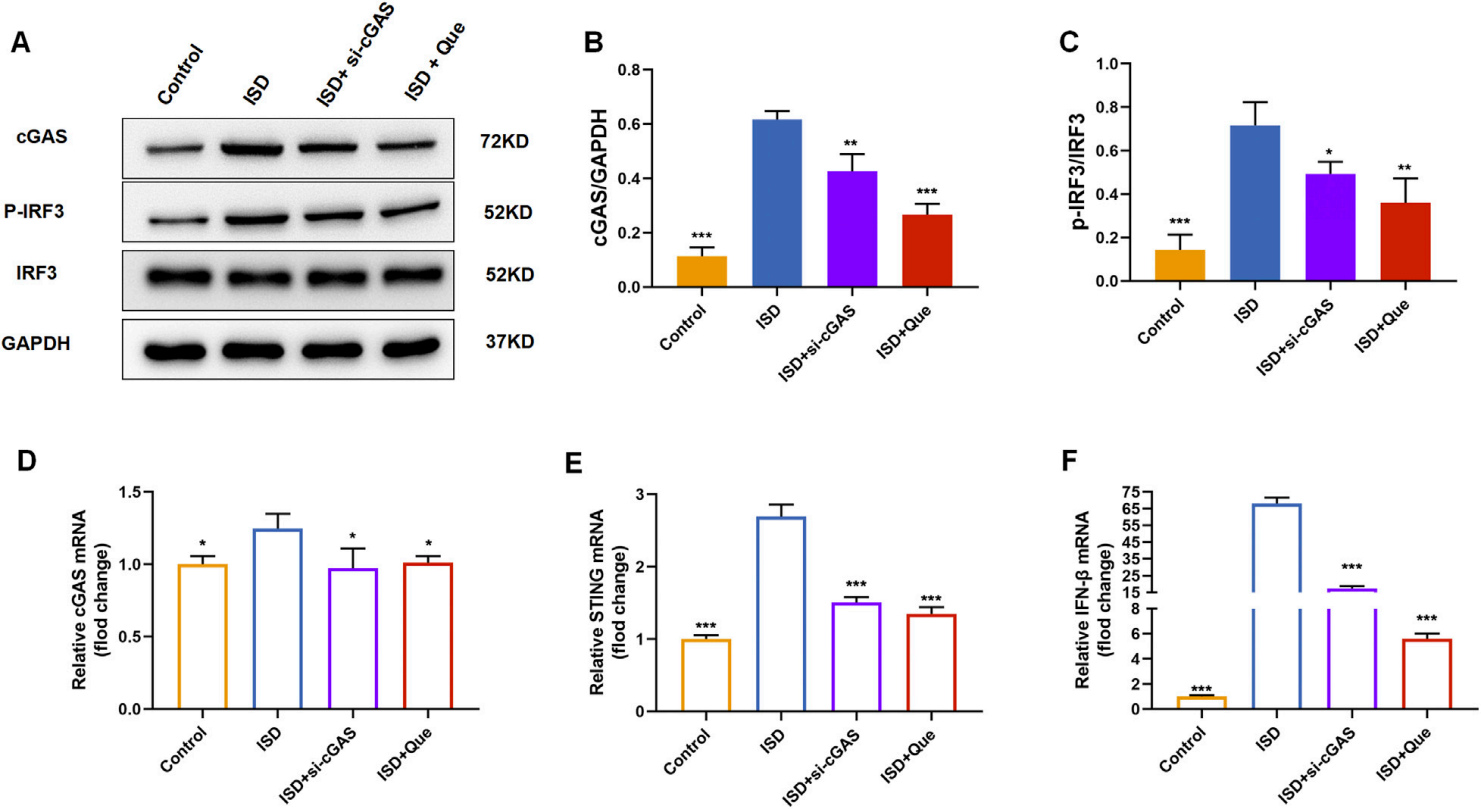
Quercetin inhibits the cGAS-STING pathway *in vitro*. **(A)** Western blot analysis of the cGAS、p-IRF3 in BMDMs (*n* = 3). **(B)** Quantification of cGAS protein levels in BMDMs. **(C)** Quantification of p-IRF3 protein levels in BMDMs. **(D–F)** Quantification of cGAS, STING, and IFN-β mRNA levels in RAW264.7 cells (*n* = 3). Data are presented as mean ± SD. *p < 0.05, **p < 0.01, ***p < 0.001.

We found that quercetin inhibited the ISD-activated cGAS-STING pathway in macrophages like RU.521 by detecting protein content and mRNA levels related to cGAS-STING pathway in BMDMs and raw264.7 cells (Figure 7). Combined with animal experiments’ results, it was indicated that quercetin’s regulation of macrophage polarization in UC mice was related to this pathway.

### 3.6 Quercetin reduces the levels of inflammation in macrophages, similar to cGAS inhibitor

**FIGURE 8:**
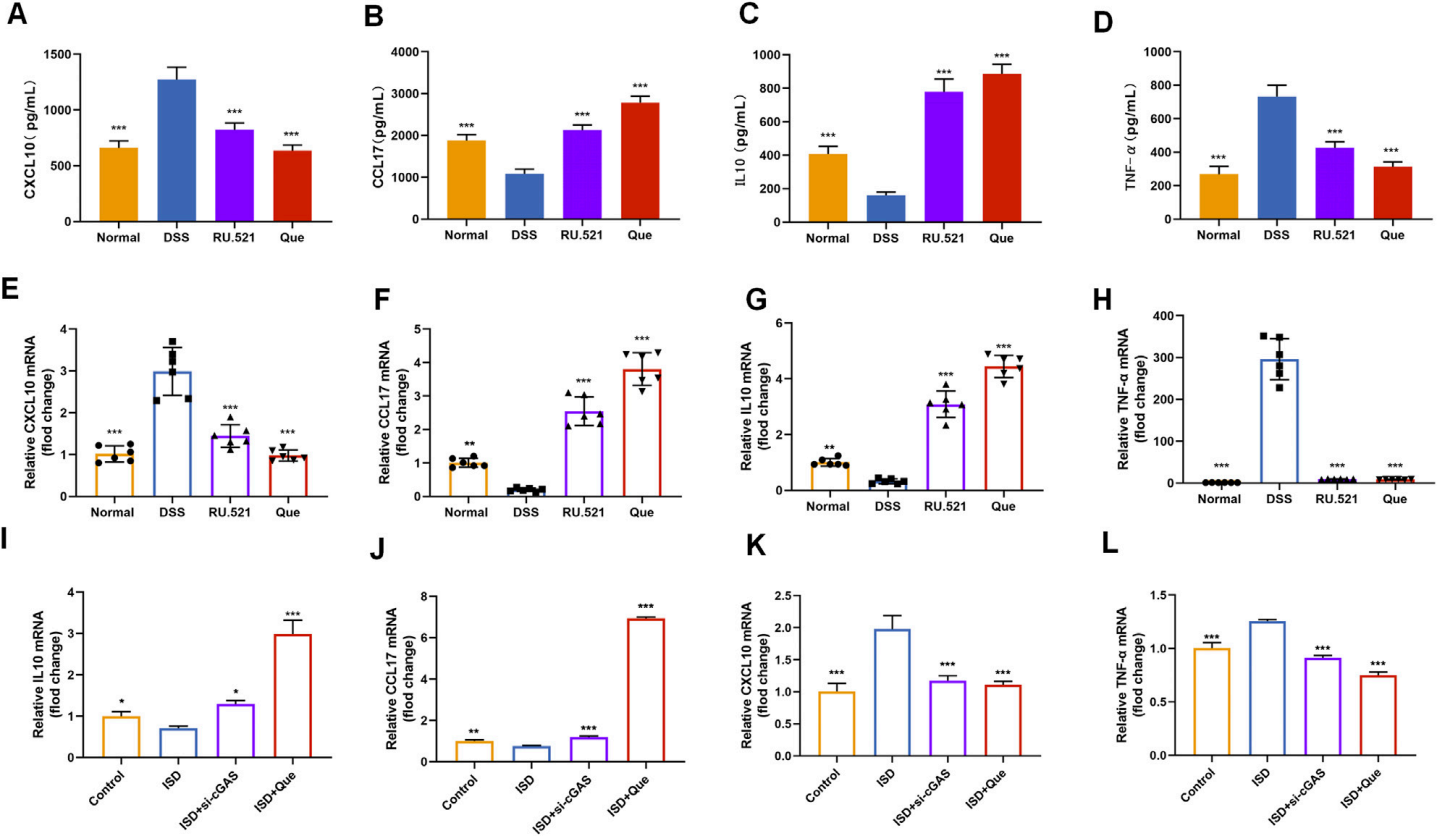
Quercetin and RU.521 reduce inflammatory factors and increase anti-inflammatory factors in colon and RAW264.7 cells. **(A–D)** Quantification of CXCL10, CCL17, IL10 and TNF-α protein levels in the colon (*n* = 6). **(E–H)** Quantification of CXCL10, CCL17, IL10 and TNF-α mRNA levels in the colon (*n* = 6). **(I–L)** Quantification of CXCL10, CCL17, IL10 and TNF-α levels in RAW264.7 cells (*n* = 3). Data are presented as mean ± SD. *p < 0.05, **p < 0.01, ***p < 0.001.

To assess the impact of Que on intestinal inflammation through the inhibition of the cGAS-STING pathway, we measured the protein levels of anti-inflammatory factors CXCL10 and TNF-α, as well as the inflammatory factors CCL17 and IL10, using ELISA. Following DSS-induced enteritis, there was a significant increase in the protein levels of CXCL10 and TNF-α, coupled with a notable decrease in IL10 and CCL17. Both Que and RU.521 could significantly reduce the protein levels of CXCL10 and TNF-α while increasing the protein levels of IL10 and CCL17 following the induction of enteritis (Figures 8A–D). Consistent results were obtained when measuring mRNA levels in intestinal tissues and macrophages (Figures 8E–L). Thus, Que could mitigate intestinal inflammation by inhibiting the cGAS-STING pathway.

### 3.7 Quercetin improves intestinal structural integrity and permeability to restore intestinal barrier functions

**FIGURE 9:**
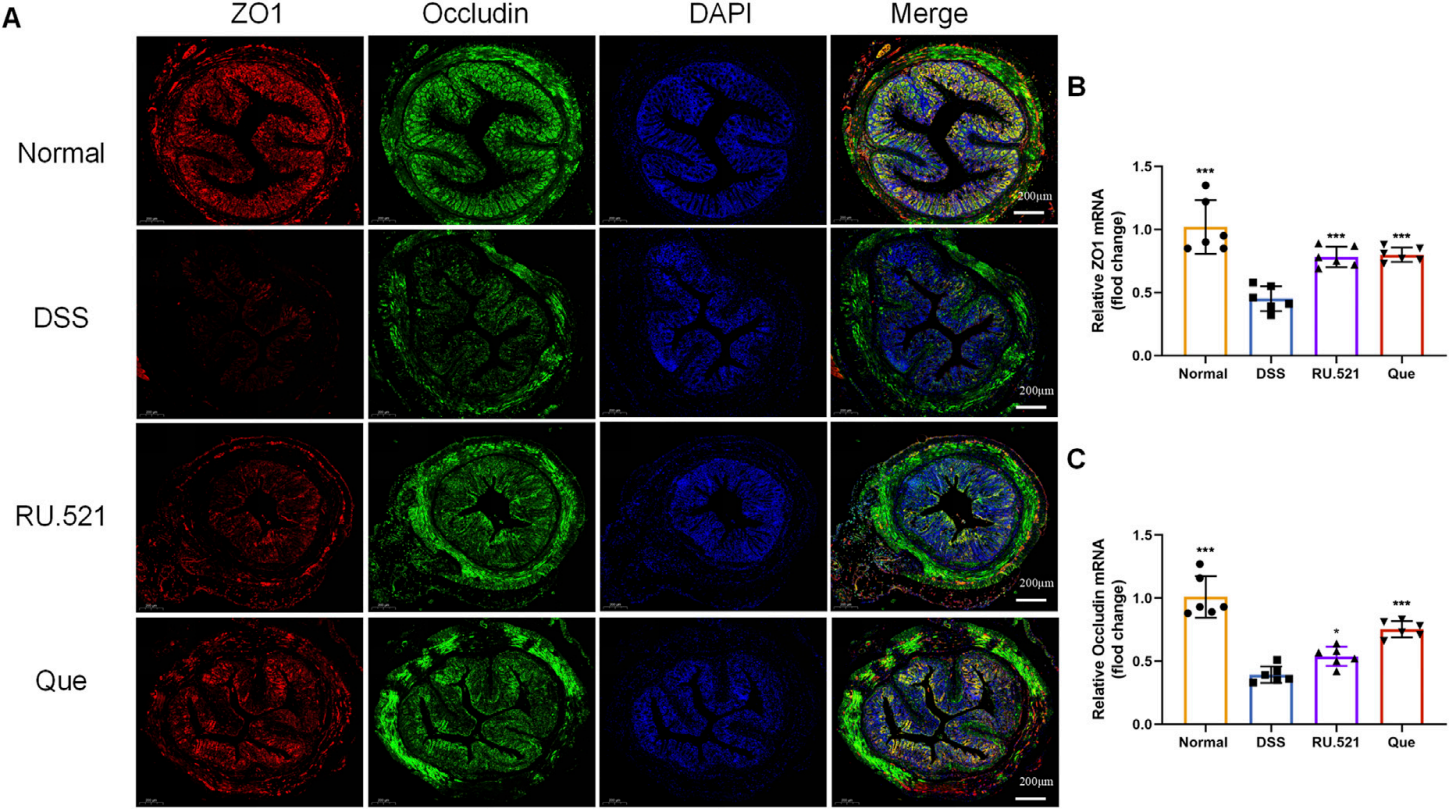
Quercetin promotes intestinal barrier function, similar to RU.521. **(A)** Representative images of ZO1 and Occludin expression examined by IF (Bar = 200 μm) in the colon. **(B,C)** Quantification of ZO1 and Occludin mRNA levels in mice colon (*n* = 6). Data are presented as mean ± SD. *p < 0.05, ***p < 0.001.

**FIGURE 10:**
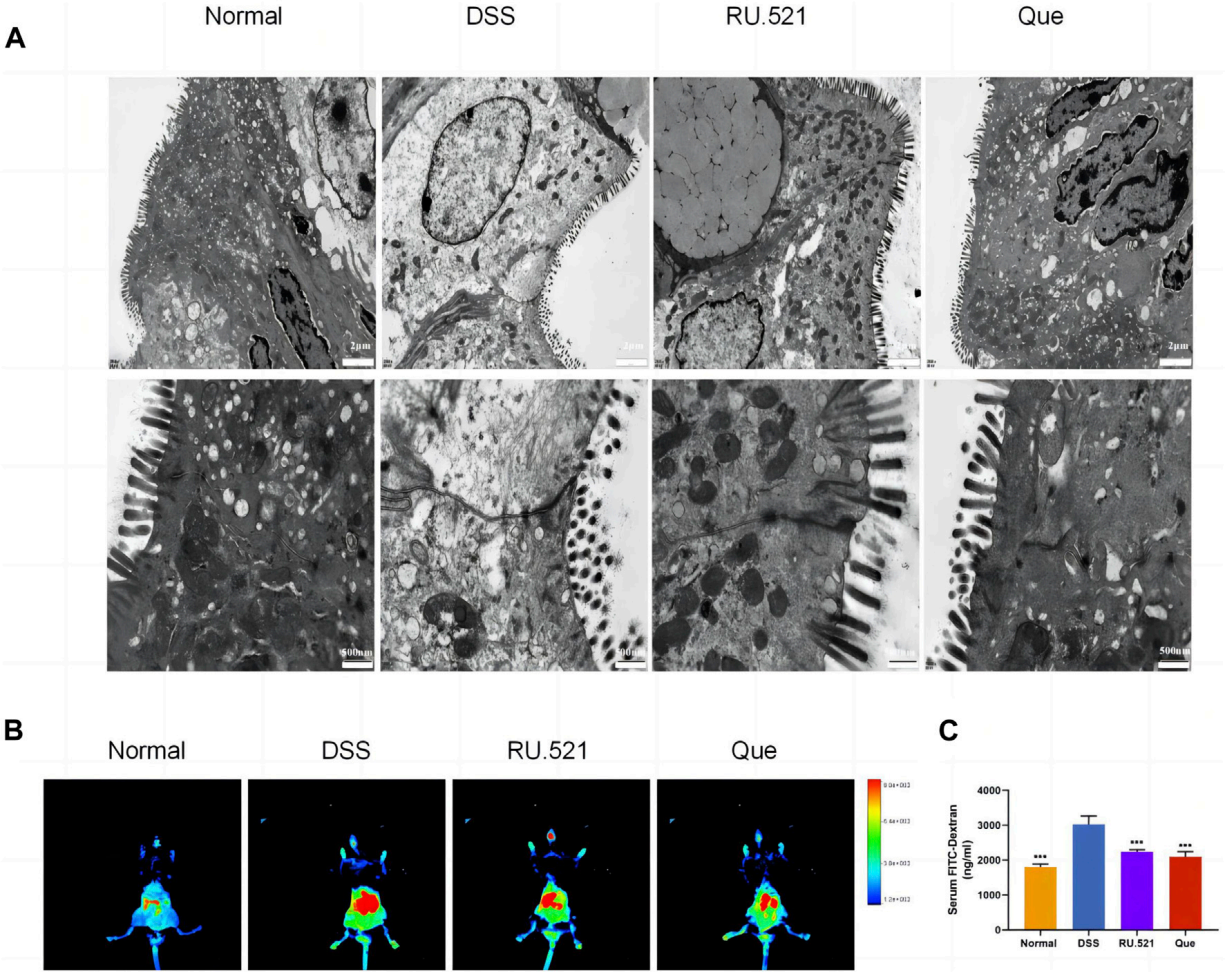
Quercetin improves intestinal structural integrity and permeability, similar to RU.521. **(A)** Representative images of intestinal ultrastructure (Bar = 2 μm, 500 nm). **(B)** Representative images of FITC-dextran distribution in mice colon. **(C)** Quantification of FITC-Dex content in mice serum (*n* = 3). Data are shown as mean ± SD. ***p < 0.001.

ZO1 and Occludin are important components of TJs that are responsible for maintaining the integrity of intestinal epithelial and endothelial cells. The structural integrity of the intestine can be visually assessed through ultrastructure analysis. In DSS-induced mice, the fluorescence expression of ZO1 and Occludin in intestinal tissues was notably weakened. However, both Que and RU.521 significantly enhanced the fluorescence expression (Figure 9A). Consistent trends were observed in the mRNA levels of intestinal ZO1 and Occludin (Figures 9B, C). In the ultrastructure analysis, normal intestinal tissues exhibited long and well-arranged microvilli, with intact TJs located directly beneath them. In contrast, the model group displayed shorter, sparser or even shed microvilli, along with interrupted TJs. Both Que and RU.521 could repair the intestinal microvilli and TJs (Figure 10A). Intestinal permeability was assessed by tracking the distribution of FITC-labeled dextran, a macromolecular compound, in the intestine and its absorption into the bloodstream. After the induction of colitis, the fluorescence distribution of FITC-Dex significantly increased, as did the content of FITC-Dex in the serum. Treatment with Que and RU.521 alleviated this situation and improved intestinal permeability (Figures 10B, C). Collectively, Que demonstrated the ability to repair the damaged intestinal structure and enhance intestinal barrier function in UC mice by inhibiting cGAS.

## 4 Discussion

**FIGURE 11:**
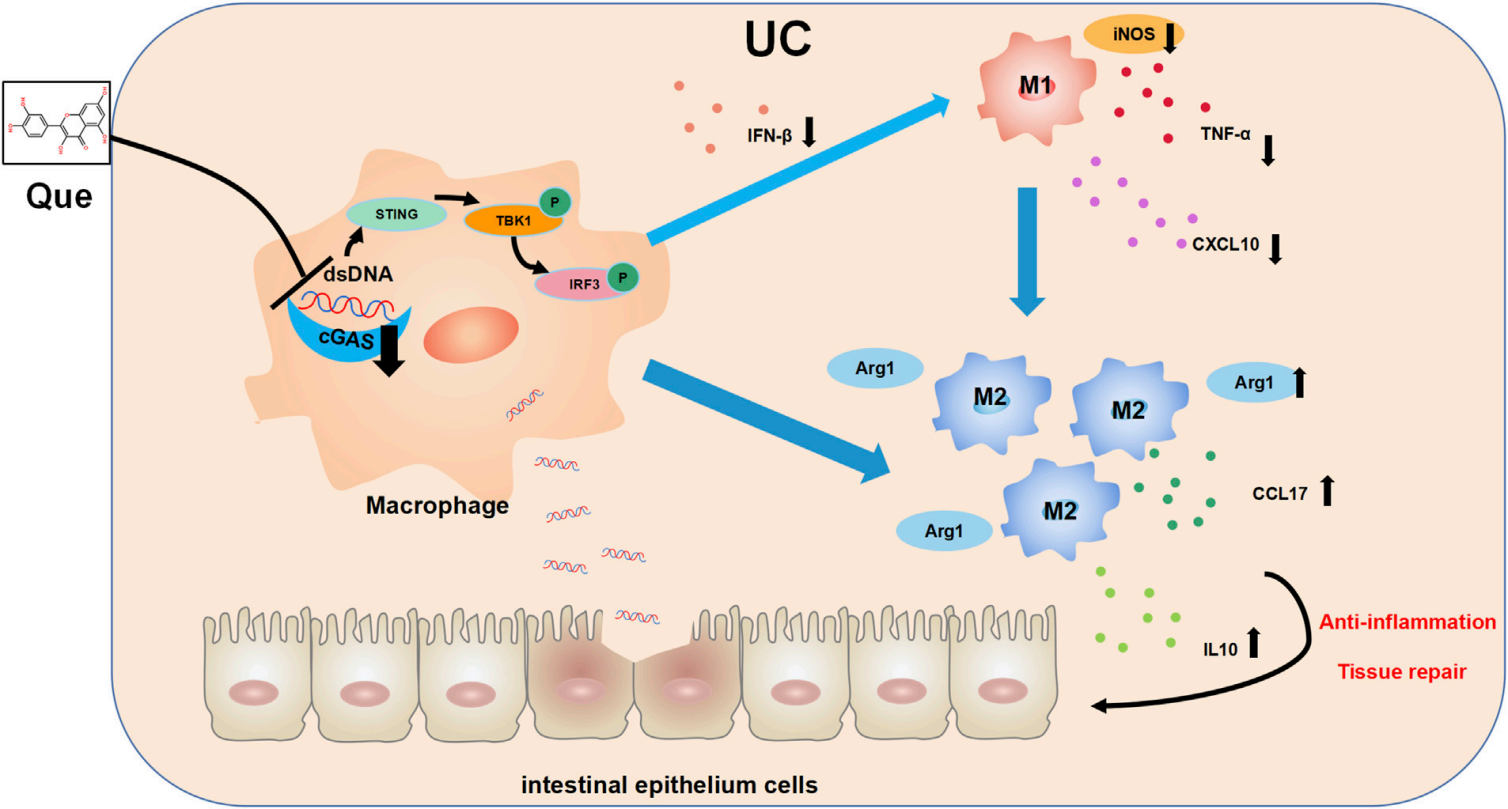
The mechanism diagram. Quercetin ameliorates ulcerative colitis by restoring the balance of M2/M1 and repairing the intestinal barrier via downregulating cGAS‒STING pathway.

In recent years, there has been growing interest in the role of imbalanced intestinal macrophage polarization in the development of UC. Que, a natural compound found in various traditional Chinese medicines, has been shown to modulate macrophage polarization and is known for its effectiveness in treating inflammatory conditions, including UC (Huang et al., 2021; Wu et al., 2022; Zhou H. F. et al., 2023). While the activation of the cGAS-STING pathway has been linked to macrophage polarization imbalance and tissue damage in the heart, its role in macrophage polarization in UC has remained unclear (Cao et al., 2018; Decout et al., 2021; Tsai et al., 2021). Distinguished from the use of LPS combined with IFN-γ as stimulants for macrophage M1 polarization, this study employed synthetic dsDNA, namely, ISD, as the stimulant in the cellular part, confirming the promotive effect of ISD on macrophage inflammatory polarization. Furthermore, Que was found to inhibit the M1 polarization induced by ISD stimulation. Additionally, in intestinal tissues, Que reduced the expression of dsDNA and inhibited M1 polarization in UC mice, which was associated with the inhibition of the cGAS-STING pathway. In this study, we have provided evidence demonstrating that Que regulates macrophage polarization triggered by dsDNA through the cGAS-STING pathway. Furthermore, Que contributed to the restoration of the intestinal barrier, leading to the alleviation of UC (Figure 11).

In this study, we first demonstrated that Que effectively alleviated UC induced by DSS in mice based on significant improvements in various key parameters, including body weight, DAI, colon length, histopathological alterations and the preservation of goblet cells, which are all important for assessing the progression of enteritis-related diseases (Rhodes et al., 1985; Goyal et al., 2014). Furthermore, we employed the cGAS inhibitor RU.521 to suppress cGAS expression in the colon and observed that it could also mitigate the development of UC, suggesting that cGAS represents a potential therapeutic target for the treatment of UC and inhibiting cGAS could be a viable approach for UC therapy. However, further investigation is required to determine whether RU.521 treatment exerts a regulatory effect on macrophage polarization similar to Que.

Macrophages are a diverse group of immune cells present in various tissues, and among them, intestinal macrophages exhibit a high degree of plasticity and versatility. Macrophage polarization exists on a continuum, with M1 and M2 macrophages representing the two ends of this spectrum. M1 macrophages are classically activated and secrete pro-inflammatory cytokines such as TNF-α and IL-6, with the typical marker being iNOS, indicating their pro-inflammatory phenotype. Conversely, M2 macrophages are alternatively activated and secrete anti-inflammatory cytokines like IL-10 and TGF-β, with Arg1 serving as a characteristic marker, signifying their anti-inflammatory phenotype (Gordon et al., 2014; Zhao et al., 2017; Shapouri-Moghaddam et al., 2018). Our study revealed that both Que and RU.521 could inhibit the polarization of M1 macrophages and reduce the secretion of iNOS in the colon, spleen and mesenteric lymph nodes of mice with UC. Simultaneously, these interventions promoted the polarization of M2 macrophages and increased Arg1 expression. We further validated these findings *in vitro* using RAW264.7 cells and bone marrow-derived macrophages (BMDMs) that were successfully induced and cultured. Que, at a concentration of 50 μmol/L, was determined to be safe for BMDMs and RAW264.7 cells and effectively corrected the imbalance in macrophage polarization induced by ISD stimulation *in vitro*. The inhibition of cGAS in macrophages also led to a reduction in M1 polarization and an increase in M2 polarization, consistent with a prior study by Cao et al. (2018).

When pathogens invade the body, both pathogen DNA and host cell DNA, which may have been damaged by the pathogen, are released into the host cell’s cytoplasm and accumulate, especially dsDNA, and represent one of the reasons for the observed abnormal increase in intestinal dsDNA levels observed in UC. At this point, cGAS, functioning as a cytoplasmic dsDNA sensor, becomes activated (Ou et al., 2021). In autoimmune and inflammatory diseases, the activation of the cGAS-STING pathway can lead to T cells differentiating into inflammatory phenotypes This results in the continual production of inflammatory factors, amplifying inflammatory responses. Furthermore, it induces macrophages to polarize towards the M1 phenotype (Martin et al., 2019; Shmuel-Galia et al., 2021). In this present study, we investigated how Que regulates the cGAS-STING pathway and found that similar to RU.521, Que could inhibit the activation of cGAS and STING, as well as the downstream phosphorylation of TBK1 and IRF3 in both intestinal macrophages and in vitro-cultured macrophages. Additionally, we observed a significant reduction in levels of IFN-β, TNF-α and CXCL10, which are associated with M1 macrophage polarization, in the colons of UC mice. Conversely, we observed an increase in levels of IL-10 and CCL17, which are associated with M2 macrophage polarization. Overall, Que can inhibit M1 macrophage polarization while promoting M2 macrophage polarization by suppressing the cGAS-STING pathway in UC.

Intestinal macrophages play a pivotal role in the immune defense of the intestine. The complex interplay between macrophage polarization, interactions with other immune cells, and the restoration of the intestinal barrier contributes significantly to the complexities of UC. Notably, co-culturing intestinal epithelial cells (IECs) with M1-type macrophages leads to disruptions in epithelial barrier integrity, resulting in the disturbance of TJ proteins and increased apoptosis of IECs. Conversely, co-culturing IECs with M2 macrophages does not elicit such detrimental effects (Lissner et al., 2015). In co-culture experiments involving macrophages and goblet cells, M2 macrophages can boost the expression of mucin-2 (MUC2) and SPDEF (a goblet cell marker gene). This enhancement of goblet cell production and mucus recovery are beneficial for colon healing following injury induced by DSS (Liang et al., 2022). To assess whether Que’s modulation of macrophage polarization impacts intestinal barrier restoration, further experiments were conducted. Our findings revealed that after Que and RU.521 interventions in DSS-induced UC mice, there was an upregulation in the expression of TJ proteins (Occludin and ZO1) in the gut. Additionally, the intestinal structure displayed enhanced integrity, including elongated villi and well-maintained TJs, along with reduced intestinal permeability. Occludin and ZO1 serve as predictive indicators of intestinal mucosal healing and the preservation of intestinal barrier function. Their levels decrease when intestinal mucosal damage occurs in UC (Shao et al., 2018). The disruption of intestinal structure and increased intestinal permeability are fundamental features of UC. These alterations result in the infiltration of pathogens, such as bacteria, into the intestinal lamina propria, subsequently triggering abnormal immune responses, including an imbalance in macrophage polarization (Na et al., 2019; Yao et al., 2019). Consequently, there exists a clear positive correlation between the equilibrium of intestinal barrier function and macrophage polarization. Que may inhibit the secretion of inflammatory factors by targeting the cGAS-STING signaling pathway to rectify imbalanced macrophage polarization, thereby contributing to the restoration of intestinal barrier function and ultimately ameliorating UC.

In summary, our study demonstrates that Que exerts its therapeutic effects in DSS-induced UC by modulating the cGAS-STING pathway to regulate macrophage polarization, resulting in an improvement in the structure and function of the intestinal barrier. These findings unveil a novel mechanism through which Que can be used to effectively treat patients suffering from UC.

## Data Availability

The original contributions presented in the study are included in the article/Supplementary Material, further inquiries can be directed to the corresponding authors.

## References

[B1] Cao D. J. Schiattarella G. G. Villalobos E. Jiang N. May H. I. Li T. (2018). Cytosolic DNA sensing promotes macrophage transformation and governs myocardial ischemic injury. Circulation 137 (24), 2613–2634. 10.1161/CIRCULATIONAHA.117.031046 29437120 PMC5997506

[B2] Costello S. P. Hughes P. A. Waters O. Bryant R. V. Vincent A. D. Blatchford P. (2019). Effect of fecal microbiota transplantation on 8-week remission in patients with ulcerative colitis: a randomized clinical trial. JAMA 321 (2), 156–164. 10.1001/jama.2018.20046 30644982 PMC6439766

[B3] Dalekos G. N. Manoussakis M. N. Goussia A. C. Tsianos E. V. Moutsopoulos H. M. (1993). Soluble interleukin-2 receptors, antineutrophil cytoplasmic antibodies, and other autoantibodies in patients with ulcerative colitis. Gut 34 (5), 658–664. 10.1136/gut.34.5.658 8504967 PMC1374185

[B4] Decout A. Katz J. D. Venkatraman S. Ablasser A. (2021). The cGAS-STING pathway as a therapeutic target in inflammatory diseases. Nat. Rev. Immunol. 21 (9), 548–569. 10.1038/s41577-021-00524-z 33833439 PMC8029610

[B5] Dicarlo M. Teti G. Verna G. Liso M. Cavalcanti E. Sila A. (2019). Quercetin exposure suppresses the inflammatory pathway in intestinal organoids from winnie mice. Int. J. Mol. Sci. 20 (22), 5771. 10.3390/ijms20225771 31744123 PMC6888448

[B6] Ding R. Li H. Liu Y. Ou W. Zhang X. Chai H. (2022). Activating cGAS-STING axis contributes to neuroinflammation in CVST mouse model and induces inflammasome activation and microglia pyroptosis. J. neuroinflammation 19 (1), 137. 10.1186/s12974-022-02511-0 35689216 PMC9188164

[B7] Gao F. Cui D. Zuo D. Shou Z. Yang J. Yu T. (2022). BMSCs improve TNBS-induced colitis in rats by inducing Treg differentiation by expressing PD-L1. Biotechnol. Lett. 44 (11), 1263–1275. 10.1007/s10529-022-03307-1 36261682 PMC9659505

[B8] Gordon S. Plüddemann A. Martinez Estrada F. (2014). Macrophage heterogeneity in tissues: phenotypic diversity and functions. Immunol. Rev. 262 (1), 36–55. 10.1111/imr.12223 25319326 PMC4231239

[B9] Goyal N. Rana A. Ahlawat A. Bijjem K. R. Kumar P. (2014). Animal models of inflammatory bowel disease: a review. Inflammopharmacology 22 (4), 219–233. 10.1007/s10787-014-0207-y 24906689

[B10] Gravina A. G. Pellegrino R. Palladino G. Coppola A. Brandimarte G. Tuccillo C. (2023). Hericium erinaceus, in combination with natural flavonoid/alkaloid and B3/B8 vitamins, can improve inflammatory burden in Inflammatory bowel diseases tissue: an *ex vivo* study. Front. Immunol. 14, 1215329. 10.3389/fimmu.2023.1215329 37465689 PMC10350490

[B11] Gui X. Yang H. Li T. Tan X. Shi P. Li M. (2019). Autophagy induction via STING trafficking is a primordial function of the cGAS pathway. Nature 567 (7747), 262–266. 10.1038/s41586-019-1006-9 30842662 PMC9417302

[B12] Hoeffel G. Debroas G. Roger A. Rossignol R. Gouilly J. Laprie C. (2021). Sensory neuron-derived TAFA4 promotes macrophage tissue repair functions. Nature 594 (7861), 94–99. 10.1038/s41586-021-03563-7 34012116

[B13] Hopfner K. P. Hornung V. (2020). Molecular mechanisms and cellular functions of cGAS-STING signalling. Nat. Rev. Mol. Cell Biol. 21 (9), 501–521. 10.1038/s41580-020-0244-x 32424334

[B14] Huang J. Zheng Y. Ma J. Ma J. Lu M. Ma X. (2021). Exploration of the potential mechanisms of wumei pill for the treatment of ulcerative colitis by network Pharmacology. Gastroenterology Res. Pract. 2021, 4227668. 10.1155/2021/4227668 PMC8714398 34970312

[B15] Jiang G. L. Yang X. L. Zhou H. J. Long J. Liu B. Zhang L. M. (2021). cGAS knockdown promotes microglial M2 polarization to alleviate neuroinflammation by inhibiting cGAS-STING signaling pathway in cerebral ischemic stroke. Brain Res. Bull. 171, 183–195. 10.1016/j.brainresbull.2021.03.010 33745949

[B16] Kim W. Jang J. H. Zhong X. Seo H. Surh Y. J. (2021). 15-Deoxy-△12,14-Prostaglandin J2 promotes resolution of experimentally induced colitis. Front. Immunol. 12, 615803. 10.3389/fimmu.2021.615803 33633749 PMC7901909

[B17] Li X. V. Leonardi I. Putzel G. G. Semon A. Fiers W. D. Kusakabe T. (2022). Author Correction: immune regulation by fungal strain diversity in inflammatory bowel disease. Nature 608 (7922), E21. 10.1038/s41586-022-05102-4 35859182

[B18] Liang L. Liu L. Zhou W. Yang C. Mai G. Li H. (2022). Gut microbiota-derived butyrate regulates gut mucus barrier repair by activating the macrophage/WNT/ERK signaling pathway. Clin. Sci. Lond. Engl. 1979 136 (4), 291–307. 10.1042/CS20210778 35194640

[B19] Lissner D. Schumann M. Batra A. Kredel L. I. Kühl A. A. Erben U. (2015). Monocyte and M1 macrophage-induced barrier defect contributes to chronic intestinal inflammation in IBD. Inflamm. bowel Dis. 21 (6), 1297–1305. 10.1097/MIB.0000000000000384 25901973 PMC4450953

[B20] Ma C. Yang D. Wang B. Wu C. Wu Y. Li S. (2020). Gasdermin D in macrophages restrains colitis by controlling cGAS-mediated inflammation. Sci. Adv. 6 (21), eaaz6717. 10.1126/sciadv.aaz6717 32671214 PMC7314554

[B21] Martin G. R. Blomquist C. M. Henare K. L. Jirik F. R. (2019). Stimulator of interferon genes (STING) activation exacerbates experimental colitis in mice. Sci. Rep. 9 (1), 14281. 10.1038/s41598-019-50656-5 31582793 PMC6776661

[B22] Murray P. J. (2017). Macrophage polarization. Annu. Rev. physiology 79, 541–566. 10.1146/annurev-physiol-022516-034339 27813830

[B23] Na Y. R. Stakenborg M. Seok S. H. Matteoli G. (2019). Macrophages in intestinal inflammation and resolution: a potential therapeutic target in IBD. Nat. Rev. Gastroenterology hepatology 16 (9), 531–543. 10.1038/s41575-019-0172-4 31312042

[B24] Ou L. Zhang A. Cheng Y. Chen Y. (2021). The cGAS-STING pathway: a promising immunotherapy target. Front. Immunol. 12, 795048. 10.3389/fimmu.2021.795048 34956229 PMC8695770

[B25] Park S. H. Kang K. Giannopoulou E. Qiao Y. Kang K. Kim G. (2017). Type I interferons and the cytokine TNF cooperatively reprogram the macrophage epigenome to promote inflammatory activation. Nat. Immunol. 18 (10), 1104–1116. 10.1038/ni.3818 28825701 PMC5605457

[B26] Rhodes J. M. Black R. R. Gallimore R. Savage A. (1985). Histochemical demonstration of desialation and desulphation of normal and inflammatory bowel disease rectal mucus by faecal extracts. Gut 26 (12), 1312–1318. 10.1136/gut.26.12.1312 2867955 PMC1433099

[B27] Sann H. Erichsen J. Hessmann M. Pahl A. Hoffmeyer A. (2013). Efficacy of drugs used in the treatment of IBD and combinations thereof in acute DSS-induced colitis in mice. Life Sci. 92 (12), 708–718. 10.1016/j.lfs.2013.01.028 23399699

[B28] Shao T. Zhao C. Li F. Gu Z. Liu L. Zhang L. (2018). Intestinal HIF-1α deletion exacerbates alcoholic liver disease by inducing intestinal dysbiosis and barrier dysfunction. J. hepatology 69 (4), 886–895. 10.1016/j.jhep.2018.05.021 PMC6615474 29803899

[B29] Shapouri-Moghaddam A. Mohammadian S. Vazini H. Taghadosi M. Esmaeili S. A. Mardani F. (2018). Macrophage plasticity, polarization, and function in health and disease. J. Cell. physiology 233 (9), 6425–6440. 10.1002/jcp.26429 PMC13482560 29319160

[B30] Shmuel-Galia L. Humphries F. Lei X. Ceglia S. Wilson R. Jiang Z. (2021). Dysbiosis exacerbates colitis by promoting ubiquitination and accumulation of the innate immune adaptor STING in myeloid cells. Immunity 54 (6), 1137–1153.e8. 10.1016/j.immuni.2021.05.008 34051146 PMC8237382

[B31] Subramanian Vignesh K. Landero Figueroa J. A. Porollo A. Caruso J. A. Deepe G. S. Jr (2013). Granulocyte macrophage-colony stimulating factor induced Zn sequestration enhances macrophage superoxide and limits intracellular pathogen survival. Immunity 39 (4), 697–710. 10.1016/j.immuni.2013.09.006 24138881 PMC3841917

[B32] Tsai C. F. Chen G. W. Chen Y. C. Shen C. K. Lu D. Y. Yang L. Y. (2021). Regulatory effects of quercetin on M1/M2 macrophage polarization and oxidative/antioxidative balance. Nutrients 14 (1), 67. 10.3390/nu14010067 35010945 PMC8746507

[B33] Virga F. Cappellesso F. Stijlemans B. Henze A. T. Trotta R. Van Audenaerde J. (2021). Macrophage miR-210 induction and metabolic reprogramming in response to pathogen interaction boost life-threatening inflammation. Sci. Adv. 7 (19), eabf0466. 10.1126/sciadv.abf0466 33962944 PMC7616432

[B34] Wang X. Xie X. Li Y. Xie X. Huang S. Pan S. (2024). Quercetin ameliorates ulcerative colitis by activating aryl hydrocarbon receptor to improve intestinal barrier integrity. Phytotherapy Res. PTR 38 (1), 253–264. 10.1002/ptr.8027 37873559

[B35] Wu J. Luo Y. Shen Y. Hu Y. Zhu F. Wu J. (2022). Integrated metabonomics and network Pharmacology to reveal the action mechanism effect of shaoyao decoction on ulcerative colitis. Drug Des. Dev. Ther. 16, 3739–3776. 10.2147/DDDT.S375281 PMC9620839 36324421

[B36] Yang T. Kong B. Gu J. W. Kuang Y. Q. Cheng L. Yang W. T. (2014). Anti-apoptotic and anti-oxidative roles of quercetin after traumatic brain injury. Cell. Mol. Neurobiol. 34 (6), 797–804. 10.1007/s10571-014-0070-9 24846663 PMC11488927

[B37] Yao D. Dong M. Dai C. Wu S. (2019). Inflammation and inflammatory cytokine contribute to the initiation and development of ulcerative colitis and its associated cancer. Inflamm. bowel Dis. 25 (10), 1595–1602. 10.1093/ibd/izz149 31287863

[B38] Ying W. Cheruku P. S. Bazer F. W. Safe S. H. Zhou B. (2013). Investigation of macrophage polarization using bone marrow derived macrophages. J. Vis. Exp. 76, 50323. 10.3791/50323 PMC3728835 23851980

[B39] Zhang M. Li X. Zhang Q. Yang J. Liu G. (2023). Roles of macrophages on ulcerative colitis and colitis-associated colorectal cancer. Front. Immunol. 14, 1103617. 10.3389/fimmu.2023.1103617 37006260 PMC10062481

[B40] Zhang Z. Zhou H. Ouyang X. Dong Y. Sarapultsev A. Luo S. (2022). Multifaceted functions of STING in human health and disease: from molecular mechanism to targeted strategy. Signal Transduct. Target. Ther. 7 (1), 394. 10.1038/s41392-022-01252-z 36550103 PMC9780328

[B41] Zhao F. Zheng T. Gong W. Wu J. Xie H. Li W. (2021). Extracellular vesicles package dsDNA to aggravate Crohn's disease by activating the STING pathway. Cell death Dis. 12 (9), 815. 10.1038/s41419-021-04101-z 34453041 PMC8397775

[B42] Zhao Q. Chu Z. Zhu L. Yang T. Wang P. Liu F. (2017). 2-Deoxy-d-Glucose treatment decreases anti-inflammatory M2 macrophage polarization in mice with tumor and allergic airway inflammation. Front. Immunol. 8, 637. 10.3389/fimmu.2017.00637 28620389 PMC5451502

[B43] Zhou H. F. Yang C. Li J. Y. He Y. Y. Huang Y. Qin R. J. (2023b). Quercetin serves as the major component of Xiang-lian Pill to ameliorate ulcerative colitis via tipping the balance of STAT1/PPARγ and dictating the alternative activation of macrophage. J. Ethnopharmacol. 313, 116557. 10.1016/j.jep.2023.116557 37142141

[B44] Zhou Y. Qian C. Tang Y. Song M. Zhang T. Dong G. (2023a). Advance in the pharmacological effects of quercetin in modulating oxidative stress and inflammation related disorders. Phytotherapy Res. PTR 37 (11), 4999–5016. 10.1002/ptr.7966 37491826

