# Supplementary material for: Quercetin ameliorates ulcerative colitis by restoring the balance of M2/M1 and repairing the intestinal barrier via downregulating cGAS‒STING pathway
Source: Front Pharmacol. 2024 May 7;15:1351538. doi: 10.3389/fphar.2024.1351538 (PMC11106451; doi:10.3389/fphar.2024.1351538)
Supplement: Supplementary file 1 [file DataSheet1.docx]

Supplementary Material

# Supplementary Figures


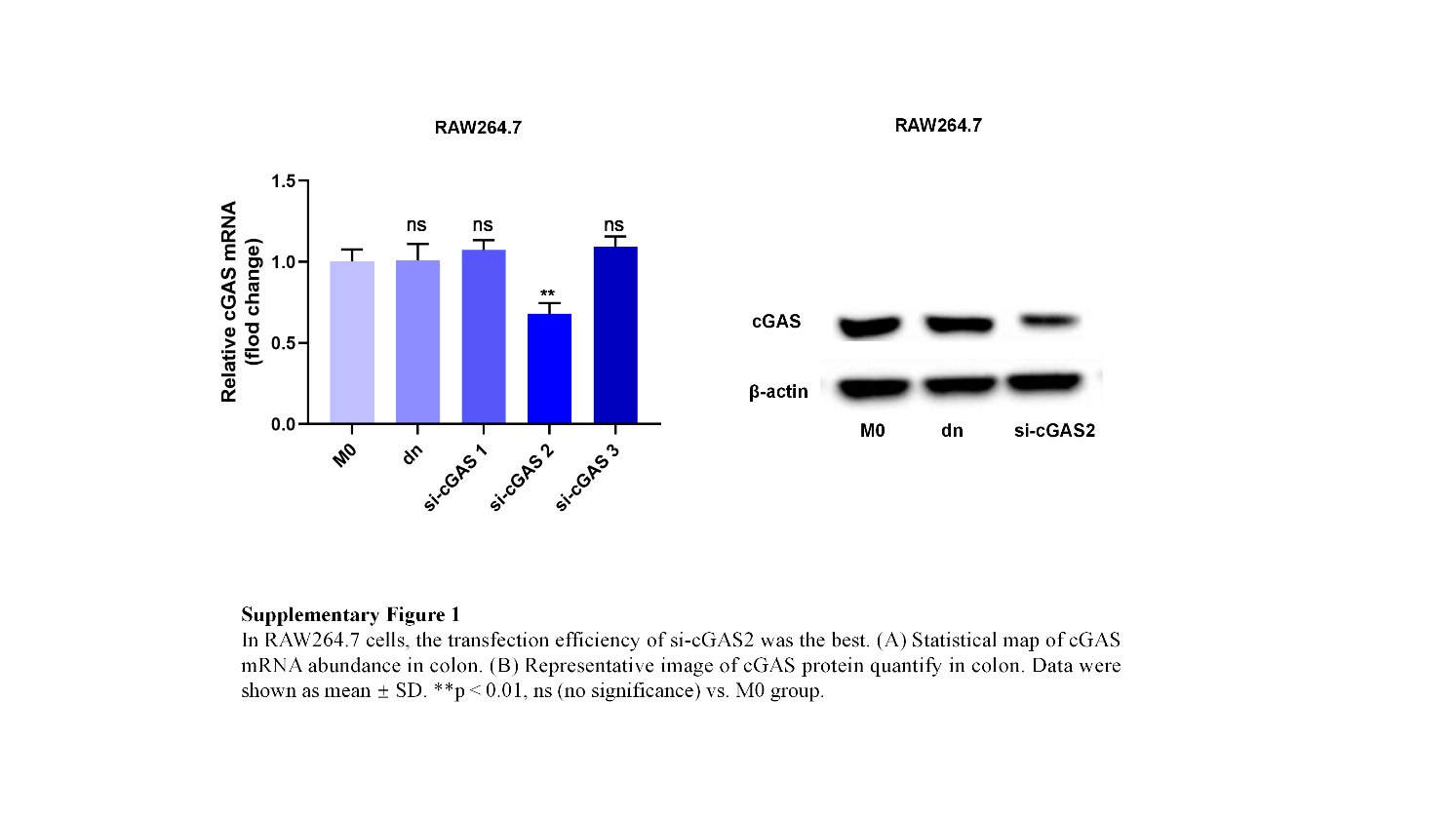


**Supplementary Figure 1.** In RAW264.7 cells, the transfection efficiency of si-cGAS2 was the best. (A) Statistical map of cGAS mRNA abundance in colon. (B) Representative image of cGAS protein quantify in colon. Data were shown as mean ± SD. **p < 0.01, ns (no significance) vs. M0 group.


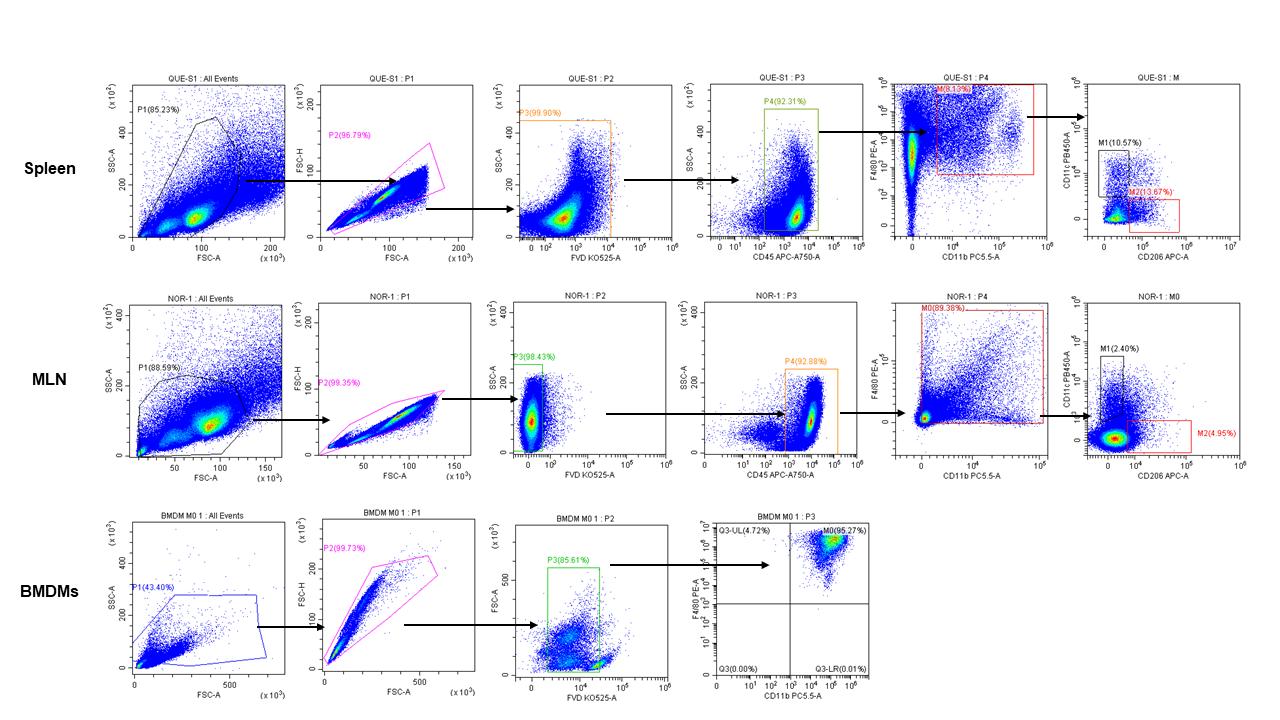


**Supplementary Figure 2.** Gating strategies of flow cytometry in BMDMs and the spleen, and MLNs.
